# A Rare Case of Native Aortic Valve Infective Endocarditis Caused by *Citrobacter koseri*

**DOI:** 10.1155/crdi/9953642

**Published:** 2026-03-16

**Authors:** Kasper Høtoft Bengtsen, Dennis Back Holmgaard, Anne-Christine Ruwald, Niels Eske Bruun

**Affiliations:** ^1^ Department of Cardiology, Zealand University Hospital, Roskilde, Denmark, regionsjaelland.dk; ^2^ Department of Clinical Microbiology, Zealand University Hospital, Køge, Denmark, regionsjaelland.dk; ^3^ Department of Cardiology, Rigshospitalet, Copenhagen, Denmark, rigshospitalet.dk; ^4^ Department of Clinical Medicine, Faculty of Health and Medical Sciences, University of Copenhagen, Copenhagen, Denmark, ku.dk; ^5^ Institute of Clinical Medicine, University of Aalborg, Aalborg, Denmark, aau.dk

## Abstract

**Background:**

Infective endocarditis (IE) is a life‐threatening condition usually caused by Gram‐positive cocci. The Gram‐negative rod *Citrobacter koseri* is an opportunistic pathogen that only rarely has been described to cause IE.

**Case Summary:**

We present a case of native aortic valve IE caused by *C. koseri* in an 84‐year‐old male. The patient was admitted three times before the diagnosis of IE was confirmed by transoesophageal echocardiography. He was initially treated with IV ceftriaxone and ciprofloxacin before undergoing open‐heart surgery with a biologic valve replacement. He was discharged 125 days after initial admission.

**Discussion:**

Presumably, the primary focus of the infection was the genitourinary tract. Despite appropriate antimicrobial therapy, infection source control was not obtained until aortic valve replacement.

**Take‐Home Message:**

The possibility of infective endocarditis should be evaluated in the case of prolonged bacteraemia with microbiological pathogens considered atypical for infective endocarditis.

## 1. Introduction

Infective endocarditis (IE) is a potentially life‐threatening infection, which requires correct and timely diagnosis and treatment to optimize outcomes. The microbiological pathogens encountered in IE are dominated by Gram‐positive cocci, while Gram‐negative microorganisms are found in a minor proportion of cases, and these are usually dominated by Gram‐negative fastidious bacteria from the *Haemophilus, Aggregatibacter, Cardiobacterium, Eikenella,* and *Kingella* (HACEK) group related to the oral–pharyngeal region [1]. *Citrobacter koseri* is a peritrichous, motile Gram‐negative rod belonging to the Enterobacterales family, commonly found in food, water, soil, and in the gastrointestinal tracts of both humans and animals [2]. *Citrobacter spp.* are opportunistic pathogens known to cause human disease, typically in the form of urinary‐tract, airway, and more rarely CNS infections, and primarily in paediatric or immunocompromised patients [3]. IE caused by *C. koseri* is rarely encountered, and only few cases have been described [4–8]. We present a case of native aortic valve IE caused by *C. koseri* in an immunocompetent 84‐year‐old Caucasian male.

## 2. Case Presentation

The patient was admitted to the emergency department of a local hospital by his general practitioner. He presented with vomiting and diarrhoea and was evaluated for infection and dehydration. His medical history included permanent atrial fibrillation (warfarin), hypothyroidism (levothyroxine), and a total prosthetic knee replacement 6 years prior to the current admission. He had no hospital admissions since the knee surgery. Prior to admission, he had a high functional level, received no nursing or domestic help, and played sports three times a week while also maintaining a small farm together with his wife. Upon arrival in the emergency department, the patient was awake and alert and neither acutely nor chronically affected. He was hemodynamically stable (BP 118/48 mmHg, pulse 68 bpm) with normal body temperature (36.8°C [98.2°F]). Physical examination revealed slight dyspnoea, an irregular pulse, and an aortic systolic murmur upon heart auscultation. His abdomen was untender with normal bowel sounds. Laboratory testing revealed a C‐reactive protein (CRP) of 240 mg/L (< 8 mg/L), a leucocyte count of 16.3 × 10^9^/L (3.5–8.8), and a neutrophil count of 14.2 × 10^9^/L (2.0–7.0). Haemoglobin (8.1 mmol/L [8.3–10.5]) and creatinine (110 [60–105]) were borderline normal. Blood and urine cultures were drawn, and the patient was admitted to the internal medicine ward for intravenous (IV) antibiotics (piperacillin/tazobactam 4 g/0.5 g q.i.d. [P/T]) and fluid replacement therapy. The next day, 3/3 blood cultures (Bact/Alert, FAN/FA aerobic and anaerobic bottles, Biomérieux) were found positive for Gram‐negative rods, which was later confirmed to be *C. koseri* identified by matrix‐assisted laser desorption ionisation time‐of‐flight mass spectrometry (MALDI‐TOF Biotyper Sirius, Bruker). Additionally, urine culture showed growth of *C. koseri* (1000 colony‐forming units/mL), both isolates sensitive to P/T interpreted using EUCAST guidelines [9] (Table 1). Inflammatory biomarkers showed a gradual reduction during the next days, but on Day 6 postadmission (PA), ciprofloxacin 500 mg b.i.d. was added to the treatment due to a lack of clinical and biochemical response. Surveillance blood cultures were drawn on Days 5, 11, 12, and 16 PA, all of which showed no microbiological growth. A transthoracic echocardiography (TTE) was performed on admission Day 9 due to suspicion of cardiac decompensation and revealed a moderate‐to‐severe aortic stenosis (max gradient 62 mmHg; Vmax 4 m/s, valve area 1.3 cm^2^ by planimetrics), moderate‐to‐severe aortic regurgitation, a mild mitral regurgitation, and normal left ventricular ejection fraction (LVEF). No additional valvular abnormalities were observed. As a further evaluation of the aortic valve, the patient was booked for a transoesophageal echocardiography (TOE), which was performed the next day and showed no signs of IE. A computed tomography (CT) scan of the thorax and abdomen was performed to ascertain whether the infection originated or had established outside of a urological focus and showed no signs of abscesses or malignancy. On Day 11 PA, the patient was afebrile but with continued elevated and stagnated inflammatory biomarkers (CRP: 85 mg/L and leucocyte count: 15.8 × 10^9^/L). After consultation with a clinical microbiologist, it was decided to stop antibiotics and draw surveillance blood cultures after a minimum of 24 h. The blood cultures showed no microbiological growth, and the patient was clinically stable and feeling well. Furthermore, a spontaneous fall in CRP and leucocyte count was observed the following days. Consequently, all antibiotics were discontinued on Day 13 PA in consultation with a clinical microbiologist. The patient was discharged feeling well on Day 19 PA with a leucocyte count of 6.9 × 10^9^/L and a CRP of 72 mg/L.

**TABLE 1:** Susceptibility testing of the isolated *C. koseri* strain.

	**First admission**	**Second admission**	**Third admission**	**MIC (μg/mL)**

Ampicillin	Resistant	Resistant	Resistant	NA
Mecillinam	Sensitive	Sensitive	Sensitive	NA
Gentamicin	Sensitive	Sensitive	Sensitive	0.5
Ciprofloxacin	Sensitive	Sensitive	Sensitive	0.016
Piperacillin/tazobactam	Sensitive	Sensitive	Sensitive	NA
Meropenem	NA	NA	Sensitive	0.032
Ceftriaxone	NA	NA	Sensitive	0.125
Cefoxitin	Sensitive	Sensitive	Sensitive	NA
Ceftazidime	Sensitive	Sensitive	Sensitive	0.125
Cefpodoxime	Sensitive	Sensitive	Sensitive	NA

*Note:* Interpretation according to EUCAST guidelines, clinical breakpoints for bacteria Version 15.0, 2025.

Two days later, he was readmitted with chills, nausea, and diarrhoea. He presented with slightly impaired consciousness (Glasgow Coma Scale 14; E4, V4, M6) but stable vital signs (BP 117/49 mmHg, pulse 81 bpm, and body temperature 37.8°C [100.0°F]). Physical examination revealed no pathological findings apart from the previously observed systolic murmur upon heart auscultation. New blood cultures were found positive for *C. koseri* (3/3), and IV P/T q.i.d. was reinstated according to susceptibility testing. The patient received nine full days of IV P/T and was discharged with additional 5 days of planned oral ciprofloxacin 500 mg b.i.d. treatment. No surveillance blood culture or echocardiography was performed.

**FIGURE 1:**
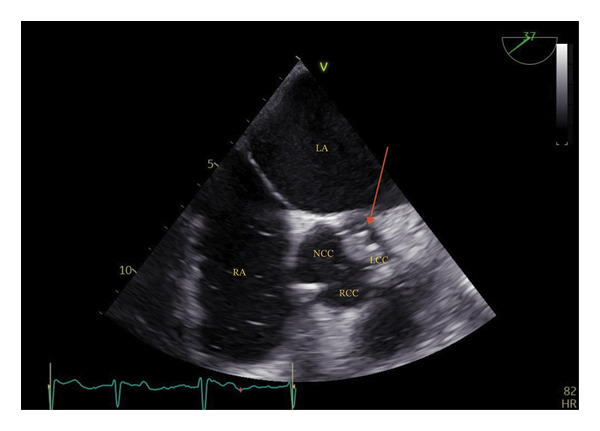
Transoesophageal echocardiography of the aortic valve showing a 10 × 10 mm vegetation located on the left coronary cusp. Red arrow indicates vegetation. Abbreviations: LA: left atrium, RA: right atrium, LCC: left coronary cusp, RCC: right coronary cusp, NCC: noncoronary cusp.

Two days after discharge, the patient was admitted for the third time to the emergency department, this time presenting with swelling of the extremities. He once again presented in a stable condition (BP 136/39 mmHg, pulse 65 bpm, and body temperature 36.5°C [97.7°F]) but with bilateral pitting oedema. Due to rising CRP (71 mg/L), IV P/T q.i.d. was reinstated but discontinued 5 days later due to lack of treatment response. Blood cultures obtained after the start of antibiotics showed no microbiological growth. Additional blood cultures were drawn 3 days after antibiotics were stopped, and for the third time, growth of *C. koseri* (3/3) was observed. IV P/T was restarted in accordance with susceptibility testing. On Day 12 from the last admission, an [18F]‐FDG positron emission tomography (PET)–CT scan was performed and showed no pathological activity, including intracardiac foci. A repeated TTE was nonconclusive for IE, and thus the patient was booked for an additional TOE which revealed a 10 × 10 mm vegetation on the left coronary cusp of the aortic valve (Figure 1) suggestive of IE, in addition to a prolapsed left cusp and moderate aortic regurgitation but preserved LVEF. The patient was transferred to an endocarditis satellite centre, and an IE antibiotic regimen consisting of IV gentamicin 3 mg/kg o.d. and meropenem 2 g t.i.d. was started. The patient was transferred to a tertiary centre for operation assessment the following day but was deemed too fragile for acute operation due to declining functional level during the admissions. He was then retransferred to the satellite centre and received a total of 29 days of conservative IE treatment with IV ceftriaxone 2 g b.i.d. and ciprofloxacin 400 mg b.i.d. before being reevaluated for operation due to persisting problems with overhydration and lack of infection control. Forty‐six days from the initiation of IE antibiotics (94 days from initial hospital admission), the patient underwent open‐heart surgery with a biological aortic valve replacement (Perimount Magna Ease, Edwards Lifesciences). At surgery, a vegetation of approx. 20 × 20 mm was located below the left coronary cusp of the native aortic valve. A temporary pacemaker was implanted postoperatively due to complete heart block, with normal sinus rhythm gradually reinstating. The temporary pacemaker was extracted after 20 days. Postextraction ECG showed intermittent atrial fibrillation/atrial flutter with normal AV‐conduction. Postoperative TTE showed a well‐functioning aortic valve prosthesis in situ and a LVEF of 35%. Anticongestive therapy was initiated, and the patient was referred to the outpatient heart failure clinic. No microorganisms could be demonstrated from microscopy of the extracted valve tissue, but *Citrobacter spp*. DNA was detected upon 16S sequencing. The endocarditis team decided on a total of 4 weeks postoperative IV antibiotics with meropenem 2 g t.i.d. and per oral ciprofloxacin 500 mg b.i.d. The patient was gradually mobilised and was discharged to his own home with outpatient rehabilitation after completed antimicrobial treatment—125 days from initial hospital admission.

## 3. Discussion

The patient described represents a rare but serious case of IE caused by non‐HACEK Gram‐negative bacteria. The case highlights the complexity and difficulties associated with the diagnostics and treatment of IE of this type. Given the positive urine culture obtained upon initial admission, it is highly plausible that the *C. koseri* bacteraemia had its origin in the genitourinary tract of the patient. *C*. *koseri* entry via the genitourinary tract is not uncommon, and an association between *C. koseri* positive urine cultures and IE has previously been described [10]. Despite IV and oral treatment with broad‐spectrum antibiotics according to susceptibility testing and multiple subsequent blood cultures without growth, the patient relapsed shortly after the first hospital discharge, raising the question of possible early valve involvement without echocardiographic signs of IE. In cases of suspected IE, echocardiographic evaluation is recommended without unnecessary delay [11]. In our case, 9 days from hospital admission to TTE might seem unnecessarily long given that the patient presented with bacteraemia and a newly discovered systolic murmur. However, IE might have seemed unlikely as *C. koseri* is a very rare cause of IE.

Furthermore, both the first TTE and TOE showed no evidence of IE. Neither did the performed CT and PET–CT scans. The 2023 ESC guidelines for the management of endocarditis [11] contain a Class I recommendation for the use of PET–CT in cases of suspected prosthetic valve IE (PVE); however, with suspected native valve IE (NVE), the sensitivity is reported around 36% [12], and thus the benefit is limited. The proposed mechanism is thought to be less frequent paravalvular involvement in NVE compared to PVE, leading to decreased inflammatory response and thus lower [18F]‐FDG uptake [11]. However, in both NVE and PVE, PET–CT can provide important information regarding extracardiac foci of infection. The PET–CT performed in our patient demonstrated no pathological [18F]‐FDG uptake. Specifically, the intracardiac activity was observed to be normal. This lack of evidence for IE is consistent with the low sensitivity of PET–CT in the setting of NVE. The patient initially received 13 full days of IV P/T with an added 7 days of per oral ciprofloxacin—a treatment which, under normal circumstances, is regarded as sufficient in the case of urosepsis given the normalization of fever and blood culture growth [13]. The isolated *C. koseri* strain was susceptible to the tested beta‐lactams except ampicillin to which it is intrinsically resistant. The bacterium retained this susceptibility throughout the period it was tested. There was no indication of ESBL or AmpC production in the isolate as it was susceptible to cefoxitin, cefpodoxime, and ceftazidime. *C*. *koseri* species harbours a chromosomal Class A beta‐lactamase that may interfere with ESBL testing [14], but in this case the isolates showed susceptibility to all cephalosporins, making the presence of ESBL highly unlikely, especially since the presence of this beta‐lactamase usually leads to overestimation of the presence of ESBL. In the same manner, the isolates showed susceptibility to ceftazidime and cefoxitin, making the presence of AmpC unlikely. The isolates also tested screening‐negative for the presence of carbapenemases with a meropenem MIC of 0.032 (screening cutoff > 0.125) [9].

The observed aortic valve abnormalities predisposed the patient to IE and could have obscured early echocardiographic signs of IE. The current ESC 2023 IE guidelines recommend IE valve surgery due to three main reasons: heart failure, uncontrolled infection, and/or high risk of embolism [11]. At the time of initial operation assessment, the patient had a Class IIa/IIb recommendation due to the risk of embolism but was deemed too fragile to undergo surgery. At the time of reevaluation 4 weeks later, both the clinical presentation (at the time, the patient was walking around the ward) and the indications for operations had changed with signs of worsening heart failure and lack of infection control.

*C*. *koseri* IE is a rare but serious condition with *Citrobacter spp.* observed in 3% of non‐HACEK Gram‐negative IE [15]. The case underscores the need for a continued search for a not remediated focus including IE, in case of lack of infection control in patients with *C. koseri* bacteraemia. Furthermore, reevaluation of indications for surgical intervention in the case of IE should be performed with due diligence. Future studies are encouraged to uncover the extent and patient implications following non‐HACEK Gram‐negative IE.

## 4. Take‐Home Messages

• The possibility of IE should be evaluated in the case of prolonged bacteraemia with microbiological pathogens considered atypical for IE.
• Reevaluation of indications for surgical intervention related to IE should be performed with due diligence.

## Funding

The authors have nothing to report.

## Consent

Written consent was obtained from the patient upon final discharge. The manuscript was presented for and approved by the patient before submission.

## Conflicts of Interest

The authors declare no conflicts of interest.

## Data Availability

Data sharing is not applicable to this article as no datasets were generated or analysed during the current study.
